# Functional integrity of visual coding following advanced photoreceptor degeneration

**DOI:** 10.1016/j.cub.2022.12.026

**Published:** 2023-01-10

**Authors:** Jessica Rodgers, Steven Hughes, Moritz Lindner, Annette E. Allen, Aghileh S. Ebrahimi, Riccardo Storchi, Stuart N. Peirson, Robert J. Lucas, Mark W. Hankins

**Affiliations:** 1 Faculty of Biology, Medicine & Health, University of Manchester, Upper Brook Street, Manchester M13 9PT, UK; 2 Nuffield Laboratory of Ophthalmology, Sleep and Circadian Neuroscience Institute, Nuffield Department of Clinical Neurosciences, University of Oxford, South Parks Road, Oxford OX1 3QU, UK; 3 Kavli Institute for Nanoscience Discovery, University of Oxford, South Parks Road, Oxford OX1 3QU, UK; 4 Institute of Physiology and Pathophysiology, Department of Neurophysiology, Philipps University, Deutschhausstr. 1-2, Marburg 35037, Germany

## Abstract

Photoreceptor degeneration sufficient to produce severe visual loss often spares the inner retina. This raises hope for vision restoration treatments using optogenetics or electrical stimulation, which generate a replacement light input signal in surviving neurons. The success of these approaches is dependent on the capacity of surviving circuits of the visual system to generate and propagate an appropriate visual code in the face of neuroanatomical remodeling. To determine whether retinally degenerate animals possess this capacity, we generated a transgenic mouse model expressing the optogenetic actuator ReaChR in ON bipolar cells (second-order neurons in the visual projection). After crossing this with the *rd1* model of photoreceptor degeneration, we compared ReaChR-derived responses with photoreceptor-driven responses in wild-type (WT) mice at the level of retinal ganglion cells and the visual thalamus. The ReaChR-driven responses in *rd1* animals showed low photosensitivity, but in other respects generated a visual code that was very similar to the WT. *ReaChR rd1* responses had high trial-to-trial reproducibility and showed sensitivity normalization to code contrast across background intensities. At the single unit level, *ReaChR*-derived responses exhibited broadly similar variations in response polarity, contrast sensitivity, and temporal frequency tuning as the WT. Units from the WT and *ReaChR rd1* mice clustered together when subjected to unsupervised community detection based on stimulus-response properties. Our data reveal an impressive ability for surviving circuitry to recreate a rich visual code following advanced retinal degeneration and are promising for regenerative medicine in the central nervous system.

## Introduction

Information processing in the central nervous system relies upon the properties of individual neurons and their arrangement into functional circuits. Alterations in these properties in degenerative conditions thus represent a fundamental challenge for regenerative medicine. As more approaches to restore function at the primary lesion site become available, an important question is the degree to which downstream neural circuits can effectively use reactivated inputs.^1^ Vision represents an excellent model system to study this problem. First, the retina and brain perform signal computations to produce a diverse and complex representation of the visual environment (the visual code), which can be assessed using carefully designed stimuli. Second, animal models of photoreceptor degeneration, such as the *Pde6b*^*rd1*^ (*rd1*) mouse, provide well-defined defects in visual input,^2–4^ which have been associated with secondary degeneration and retinal circuit reorganization.^5–7^ Finally, pre-clinical methods of vision restoration are well established.^8–10^ Here, we aim to exploit these advantages and determine the capacity of visual circuits in mice with advanced retinal degeneration to generate an intact visual code.

Our approach was to use the optogenetic actuator ReaChR^11^ to activate the first post-receptor neurons (ON bipolar cells [OBCs]) in the visual projection of *rd1* mice, allowing the functional capacity of the surviving retinal circuitry to be probed by measuring ReaChR-driven responses in downstream neurons. Directly photosensitizing OBCs using optogenetics has consistently been shown to restore visual responses in retinally degenerate mice using electrophysiology and behavioral testing.^12–18^ Downstream neurons in treated *rd1* mice can resolve differences in brightness and rapidly respond to visual stimuli (albeit at higher light levels than wild types [WT]). These are encouraging outcomes but do not address perhaps the most fundamental feature of visual circuits—the ability to parse visual scenes into parallel information pathways with different feature selectivity.

The precise content and number of retinal information channels vary with classification method.^19–21^ Baden et al.^22^ used two-photon calcium imaging of mouse retinal ganglion cells (RGCs) combined with standardized stimuli designed to test key characteristics—including response polarity, contrast sensitivity, temporal frequency tuning, color opponency, direction selectivity, and receptive field size. Unsupervised clustering of functional responses, combined with cell morphology, revealed 32 output channels. Recently, an equally comprehensive study found as many as 45 RGC types in the mouse retina.^23^ This diversity is preserved in the dorsal lateral geniculate nucleus (dLGN), a key retino-recipient brain area in transmitting visual information to the primary visual cortex, where responses reflect the activity of either individual or heterogeneous combinations of different RGC types.^24–26^

The capacity of late-stage degenerate retinas, following profound rod and cone loss,^7^ to support a rich visual code remains largely unexplored. Some aspects of response diversity in *rd1* retinas during vision restoration studies have been indirectly assessed. Thus, following optogenetic photosensitization of OBCs, some groups report both ON and OFF responses^13,15,17,18^ in *rd1* RGCs, whereas others find an absence of OFF responses.^14,16,27^ There are more reliable reports of variation in response transience to a light step.^27^ However, a challenge in interpreting these data is that the method used to express optogenetic activators (recombinant viral gene delivery) provides different levels of expression across the target cell population, imposing potential stochastic variation in the response properties of downstream neurons.

We overcome this issue by generating a transgenic *rd1* mouse for more uniform *ReaChR* expression in OBCs. Using a battery of stimuli, we compare the visual response properties of *ReaChR rd1* mice with those driven by rods and cones in WT mice. Recording from the retina and dLGN, we find remarkable concordance between the two groups, from fundamental features such as contrast sensitivity, temporal frequency tuning, and response reproducibility to overall visual code complexity. Our work provides hope that neural circuits can appropriately process and transmit information even following advanced degeneration.

## Results

### Optogenetic interrogation of retinal circuits

**Figure 1:**
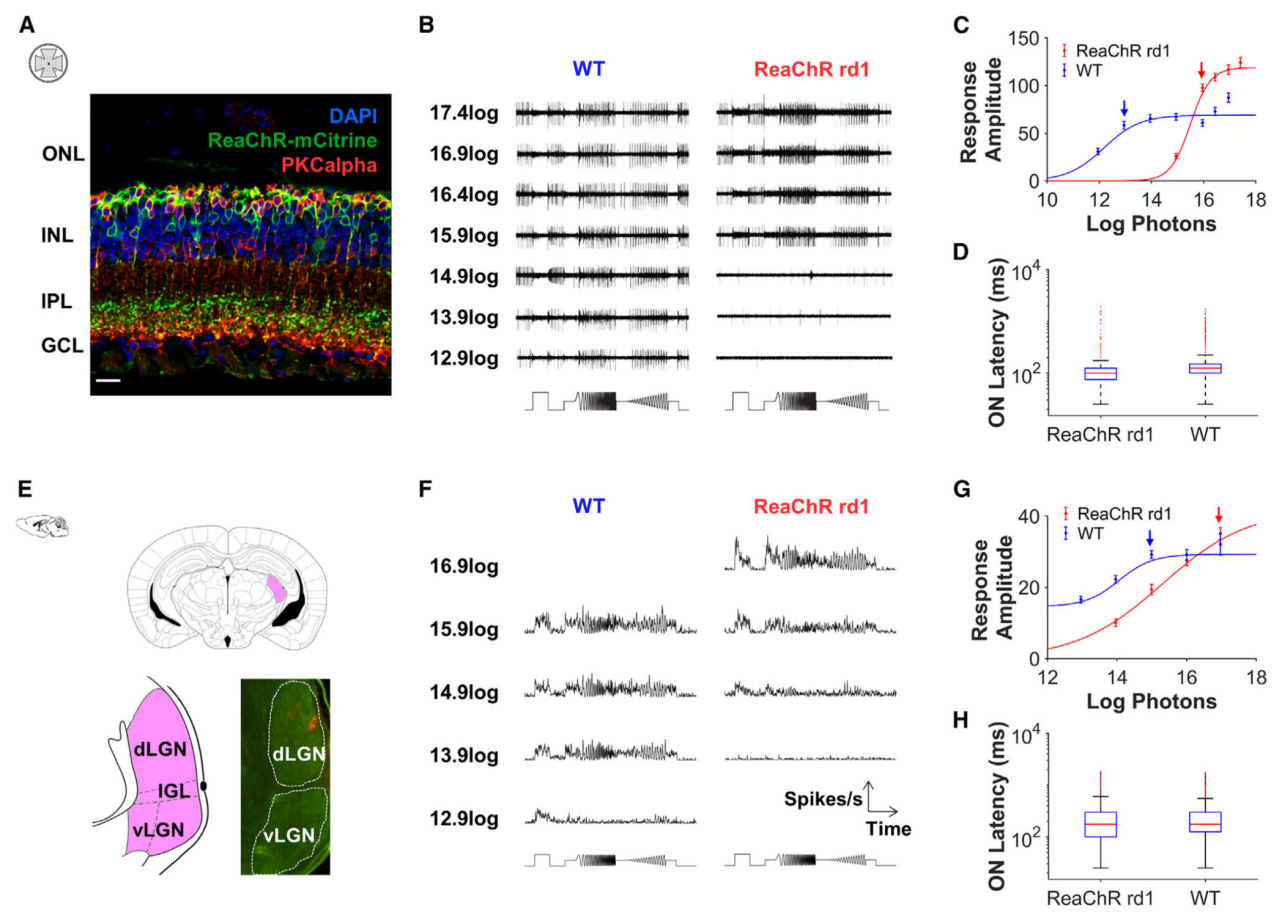
Optogenetic interrogation of *rd1* retinal circuits (A) *ReaChR rd1* retinal sections co-stained for *ReaChR-mCitrine* (green), PKCα (red), and DAPI (blue). Outer nuclear layer (ONL), inner nuclear layer (INL), inner plexiform layer (IPL), and ganglion cell layer (GCL) are labeled. Scale bar, 20 mm. (B) Representative responses (raw data) from MEA recordings of *ReaChR rd1* and WT retinal explants across irradiances. (C and G) Irradiance response curves for transient and sustained ON units from (C) retina (*ReaChR rd1* n = 414, WT n = 289) and (G) dLGN (*ReaChR rd1* n = 225, WT n = 258) units. (D and H) Response latency to onset of 3 s step for ON transient, sustained, and biphasic light-responsive units from (D) retina (*ReaChR rd1* n = 661, WT n = 841) and (H) dLGN (*ReaChR rd1* n = 329, WT n = 352) units. (E) dLGN recordings. LGN is shown in pink on the coronal brain section (top) with details of dorsal LGN (dLGN), ventral LGN (vLGN), and intergeniculate leaflet (IGL) on the bottom left. The bottom right shows the histology of dLGN, with electrodes placement in red. Brain diagrams adapted from Paxinos and Franklin.^30^ (F) Representative PSTH (25 ms bins) showing responses from *ReaChR rd1* and WT dLGN unit across irradiances. Stimulus timing shown below (B) and (F). Irradiances used for comparison between groups are marked with arrows in (C) and (G). Error bars show SEM. See also Figure S1.

We developed a transgenic mouse, *ReaChR-mCitrine; Grm6*^*cre*^; *Pde6b*^*rd1/rd1*^ (*ReaChR rd1* throughout), that expresses ReaChR in OBCs, the earliest surviving neurons in the *rd1* visual pathway. By measuring ReaChR-driven responses in downstream neurons, we can probe signal processing in the degenerate retina. ReaChR produces reproducible responses with high temporal resolution,^11,28^ allowing us to explore the true functional limits of the *rd1* retina. We confirmed the *Grm6*^*cre*^*-*driver line^29^ results in *ReaChR* expression in OBCs by co-localization of *ReaChRmCitrine* fluorescent tag with staining against the rod bipolar cell marker, PKCalpha (Figures 1A and S1).

### Light sensitivity, response amplitude, and latency

**Figure 2:**
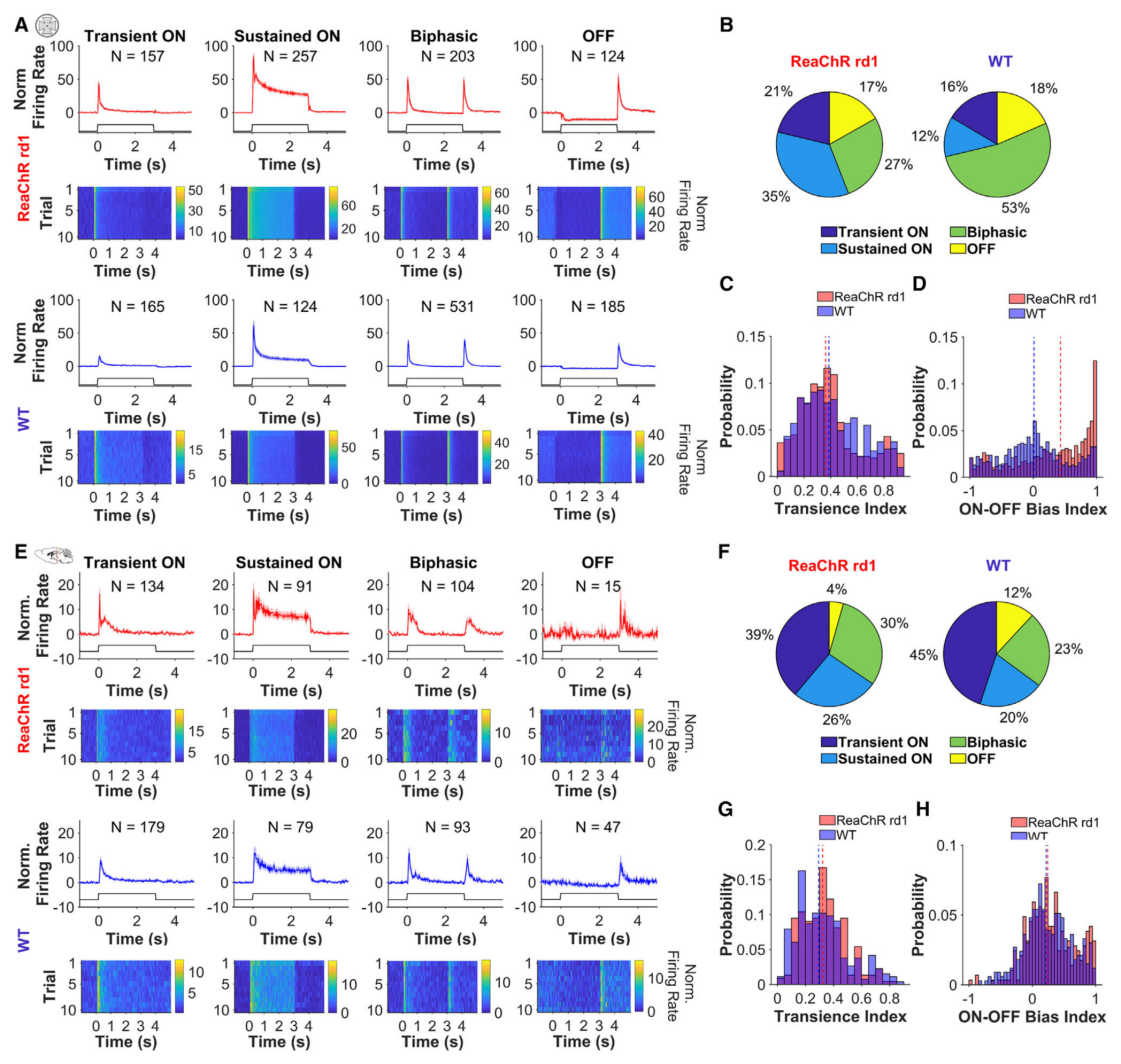
Responses to step stimuli (A and E) Mean PSTH (25 ms bins, top and third row) and heatmap of mean response across trials (second and bottom row) in response to 3 s light step (onset at 0 s) for 4 different response categories detected in *ReaChR rd1* (top two rows) and WT (bottom two rows) in (A) retina and (E) dLGN. Error bars show SEM. (B and F) Pie chart showing the distribution of units across 4 different response categories in (B) retina and (F) dLGN. (C and G) Histogram of transience index for light-responsive (LR) units in (C) retina (*ReaChR rd1* n = 605, WT n = 509) and (G) dLGN (*ReaChR rd1* n = 221, WT n = 264). (D and H) Histogram of ON-OFF bias index for LR units in (D) retina (n = 682 for *ReaChR rd1*, n = 765 for WT) and (H) dLGN (*ReaChR rd1* n = 286, WT n = 332).

**Figure 3:**
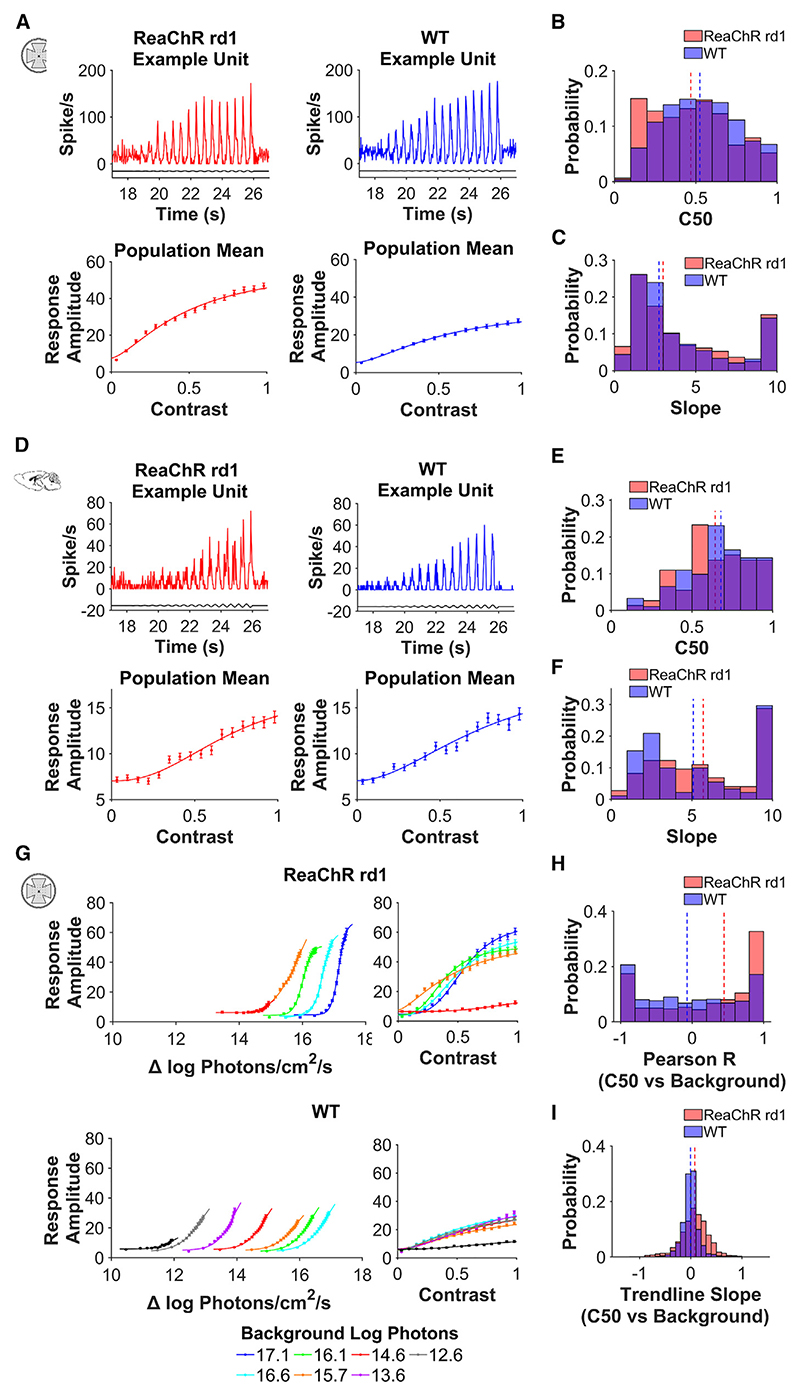
Contrast sensitivity (A and D) Top panel shows representative PSTH (25 ms bin) in response to contrast chirp for individual (A) retina and (D) dLGN units. Timing of the contrast chirp stimulus shown in black. Bottom panel shows the contrast sensitivity function for the population mean from (A) retina and (D) dLGN units. (B, C, E, and F) Histogram of (B and E) C50 and (C and F) slope derived from best-fit contrast sensitivity function in (B and C) retina (*ReaChR rd1* n = 440, WT n = 476) and (E and F) dLGN (*ReaChR rd1* n = 73, WT n = 91). (G) Response amplitude to contrast chirp as a function of Δirradiance (left) and Michelson contrast (right) over different background irradiances for *ReaChR rd1* (n = 178–741) and WT (n = 327–1,228) in the retina. (H and I) Histogram of (H) Pearson R and (I) slope of linear trendline for C50 against background irradiance for *ReaChR rd1* (n = 636) and WT (n = 1,309) retinal units. The dashed lines in (B), (C), (E), (F), (H), and (I) show the median. Errors bars show SEM. See also Figure S2.

**Figure 4:**
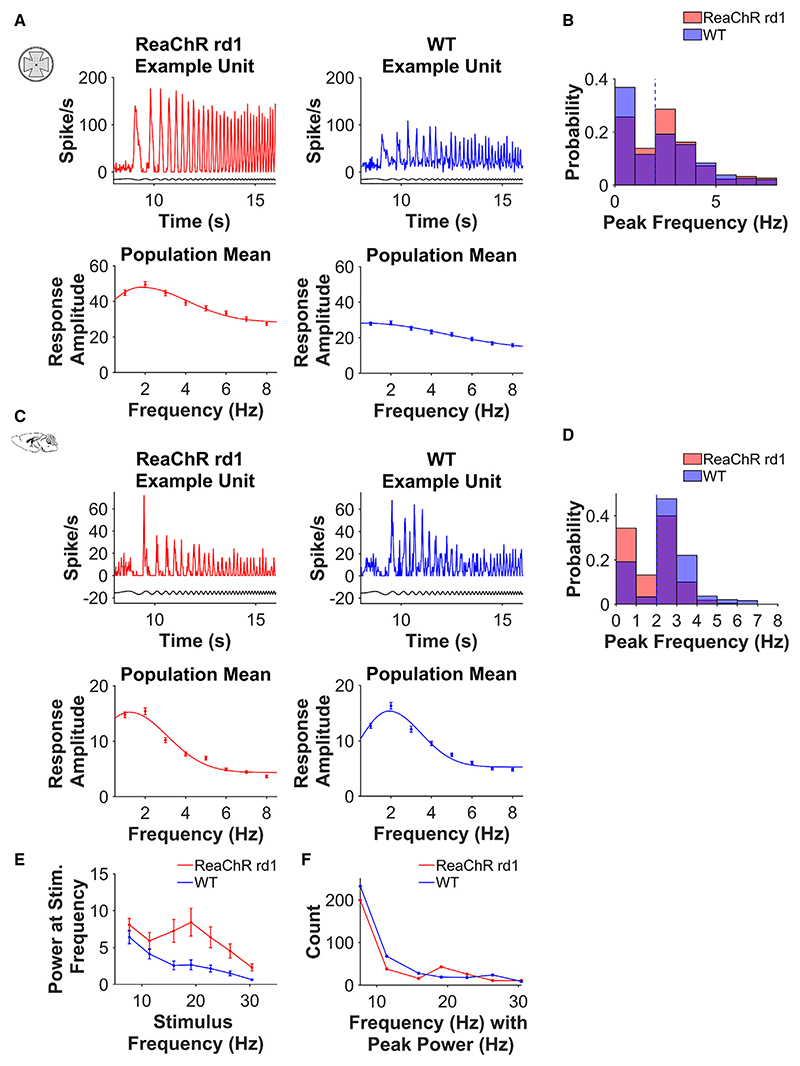
Temporal frequency tuning (A and C) Top panel shows representative PSTH (25 ms bin size) in response to temporal chirp for individual (A) retina and (C) dLGN units. Timing of the contrast chirp stimulus shown in black. The bottom panel shows the temporal frequency tuning function for the population mean from (A) retina and (C) dLGN units. Timing of the chirp stimulus shown in black. (B and D) Histogram of peak temporal frequency (rounded to the nearest integer) derived from best-fit temporal frequency tuning function from (B) retina (*ReaChR rd1* n = 518, WT n = 619) and (D) dLGN (*ReaChR rd1* n = 218, WT n = 239) units. (E) Mean power at different temporal frequencies (calculated from fast-Fourier transform) for light-responsive units (*ReaChR rd1* n = 344, WT n = 398) in dLGN. (F) Distribution of dLGN units with peak power at each stimulus frequency. The dashed line in (B) and (D) shows the median. Error bars show SEM. See also Figures S3 and S4.

**Figure 5:**
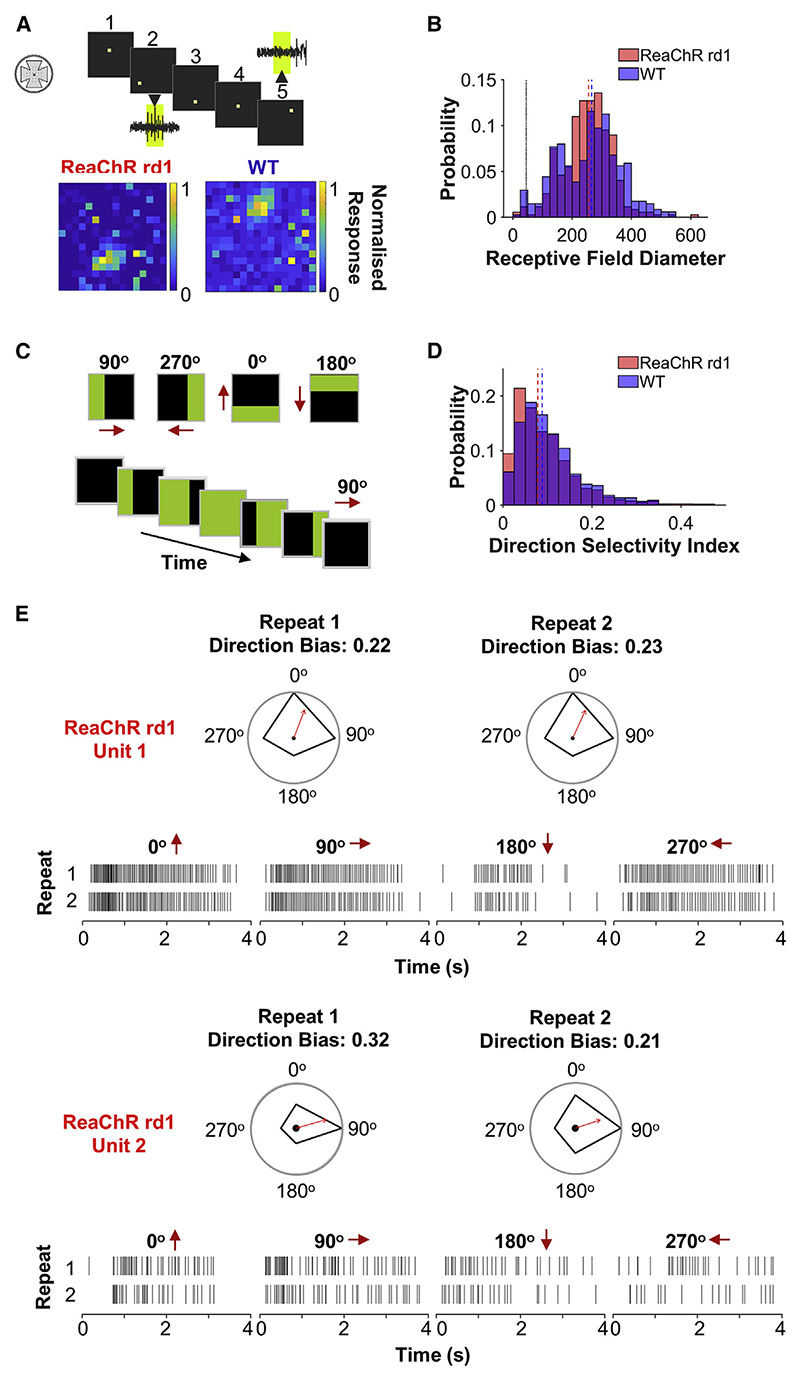
Receptive field mapping and direction selectivity (A) Receptive field maps were obtained by recording activity in response to different positions of a light spot in a 16 × 16 grid (examples shown for positions 2 and 5). Stimuli schematic from Lindner et al.^27^ Below are example receptive fields for *ReaChR rd1* and WT retinal ganglion cells. (B) Histogram of receptive field diameter (µm) for light-responsive units from *ReaChR rd1* (n = 346) and WT (n = 339) retinas. The dotted black line shows the size of the spot stimulus (~42 µm). (C) Direction selectivity was based on response to the motion of a 565 nm bar in 4 directions (0°, 90°, 180°, and 270°) shown on top. An example of the motion of the bar in 90° direction shown on bottom. Red arrows show the direction of the moving bar. (D) Histogram of the direction selectivity index for light-responsive units from *ReaChR rd1* (n = 392) and WT (n = 441) retinas. (E) Raster plots for two direction-selective units found in the *ReaChR rd1* retinas and polar plot of preferred direction. Two repeats are shown for each direction of the moving bar, with the resulting polar plot of each trial. Dashed lines in (B) and (D) show the median.

To define the functional capacity of *rd1* visual circuits, we presented a full-field “chirp” stimulus^22^ (Figure 1B) to 5-month-old WT and *ReaChR rd1* mice. We separately recorded responses from RGCs (*ex vivo* retinal explants) or dLGN neurons (*in vivo* under anesthesia). Although we found responses to this stimulus across all light intensities tested in WT mice, only the brighter stimuli elicited responses in *ReaChR rd1* animals (Figures 1B and 1C).

We first quantified the change in firing rate to the first stimulus element, the 3 s light step from the dark. In the retina, 77% of *ReaChR rd1* and 81% of WT units responded to this step at 16.95 log photons/cm^2^/s. At this intensity, *ReaChR rd1* retinal units showed a larger amplitude response than WT (maximum baseline-normalized firing rate median for *ReaChR rd1* = 93.9 spikes/s, WT = 70.9 spikes/s, U = 5.70, p < 0.001). To avoid issues of response saturation, subsequent comparisons between genotypes were performed at sub-saturating (75% response amplitude) irradiances (15.9 and 12.9 log photons/cm^2^/s for *ReaChR rd1* and WT, respectively). *ReaChR rd1* retinas showed reduced response latency under these sub-saturating conditions (Figure 1D; median latency = 0.1 s for *ReaChR rd1*, 0.125 s for WT, U = −13.7, p < 0.001).

Units showing responses to the brightest light step were also common in the dLGN (Figure 1E) of both genotypes (67% *ReaChR rd1*, 60% WT units at 16.97 log photons/cm^2^/s; Figure 1F). As in the retina, dLGN responses driven by *ReaChR* were restricted to higher irradiances (Figure 1G). The maximal response amplitude was larger for *ReaChR rd1* compared to WT (median = 27.6 and 23.8 spikes/s, respectively, U = 3.22, p = 0.001), although dLGN firing rates were markedly lower compared with the retina. As above, we identified sub-saturating intensities for *ReaChR rd1* and WT dLGN responses (16.97 log and 14.99 log photons/cm^2^/s, respectively). Under these conditions, as in the retina, *ReaChR*-driven dLGN light responses showed reduced response latency (median = 0.18 s, range = 0.03–1.83 s for *ReaChR rd1*, median = 0.18 s, range = 0.03–1.75 s for WT, U = −3.46, p < 0.001; Figure 1H).

### Step response categories

The most fundamental distinctions in visual response properties are between cells activated by increases and/or decreases in light intensity (ON vs. OFF), and those with either sustained vs. transient responses to a light step. This diversity was clearly retained in *ReaChR rd1* retina and dLGN responses to the 3 s step (Figures 2A and 2E). Systematic criteria were used to place each light-responsive unit into one of four response categories: transient ON, sustained ON, OFF, and biphasic ON-OFF. Importantly, all categories found in the WT retina were also present in the *ReaChR rd1* retina (Figure 2B). Although the proportion of transient ON and OFF responses was similar between genotypes, the *ReaChR rd1* retina showed more sustained ON responses and fewer biphasic responses compared with WT (Figure 2B). For a more quantitative comparison, we calculated indices for transience (1 = highly sustained) and ON-OFF bias (−1 = OFF, 1 = ON) for all units.^20^ Response transience was comparable for *ReaChR rd1* (median = 0.36, range = 0.02–0.94) compared with *WT* (median = 0.39, range = 0.03–0.93; U = −1.64, p = 0.102; Figure 2C). However, *ReaChR rd1* units were significantly more biased toward ON responses (median = 0.43) compared with WT (median = 0.01, U = 9.21, p < 0.001; Figure 2D).

At the level of the dLGN, the relative distribution of units across response categories was generally comparable between *ReaChR rd1* and WT (Figure 2E), with fewer OFF units in both genotypes compared with the retina (Figure 2F). The distribution of ON-OFF bias index values showed *ReaChR rd1* units in the dLGN were marginally more ON-biased, although this difference was not significant (median = 0.24 for *ReaChR rd1*, 0.21 for WT, U = 1.50, p = 0.133; Figure 2H) and that both groups lacked the subpopulation of strongly OFF-biased units seen in the retina. The transience index was comparable between genotypes (median = 0.29 for WT, 0.32 for *ReaChR rd1*, U = 1.33, p = 0.182; Figure 2G).

### Contrast sensitivity and normalization

To assess whether the degenerate retina can encode visual contrast, we next turned to the “contrast chirp” (2 Hz sinusoidal modulation at increasing contrast). Retinal units in both genotypes tracked this stimulus element (Figures 3A and S2). The larger response amplitude in *ReaChR rd1* was also observed for the contrast chirp (normalized amplitude at highest contrast, median = 32.0 for *ReaChR rd1*, 15.8 for WT, U = 10.28, p < 0.001). Importantly, the contrast-response functions were otherwise broadly similar between genotypes (Figure 3A).

For both *ReaChR rd1* and WT, there was substantial diversity in both the contrast that elicited a half-maximal response (C50; Figure 3B) and the slope ofthe contrast-response function (Figure 3C). Some units responded only to high contrast, whereas others tracked the entire contrast range (Figure S2). Across the population, C50 was significantly lower for *ReaChR rd1* compared to WT (median = 47% Michelson contrast for *ReaChR rd1*, 53% for WT, U = −3.35, p = 0.005), but there was no significant difference in slope (median slope = 2.99 for *ReaChR rd1*, 2.74 for WT, U= −1.77, p = 0.078). We found similar variation in contrast sensitivity between dLGN units of both genotypes (Figures 3D–3F), with no significant difference between *ReaChR rd1* and WT in either C50 or slope (median C50 = 64% for *ReaChR rd1*, 68% for WT, U= −0.78, p = 0.432; median slope = 5.71 for *ReaChR rd1*, 5.10 for WT, U = −1.56, p = 0.119).

The ability to encode contrast irrespective of background light intensity is a key characteristic of the visual system. To determine whether *ReaChR rd1* retinas retained this capacity, we compared mean contrast-response relationships across background irradiances. *ReaChR rd1* responded to contrast over an ~2 log range of background light intensities (Figure 3G). Across this range, the amplitude of modulations in ReaChR-driven firing rates was clearly separated when expressed with reference to the actual intensity of chirp components (Figure 3G, top) but was more equivalent when plotted as a function of visual contrast (Figure 3G, bottom). This property suggests that across the RGC population, response amplitude is well explained by contrast across variations in the background.

To determine whether this was also true at a single unit level, we asked how well contrast sensitivity (defined by C50) was retained across background light intensity for each unit. In the case of perfect sensitivity normalization, C50 should be fixed irrespective of background irradiance. In practice, we found there tended to be a stronger relationship between C50 and irradiance in *ReaChR rd1* compared with WT (median Pearson R = 0.45 and −0.07, respectively; Figure 3H), indicating normalization was less efficient in *ReaChR rd1*. However, the slope of this relationship was relatively shallow for *ReaChR rd1* (median slope = 0.08, WT median slope = −0.01; Figure 3I), indicating that the magnitude of the change in contrast sensitivity across background irradiance in *ReaChR rd1* was relatively small. These analyses indicate that sensitivity normalization is occurring in the *ReaChR rd1* retina to allow effective contrast coding, albeit over a relatively narrow range of background irradiances.

### Temporal frequency tuning

We next turned our attention to the “temporal chirp” (accelerating sinusoidal modulation at 97% Michelson contrast). Both genotypes showed strong responses to this stimulus element in the retina (Figures 4A and S3), with individual units showing variability in preferred temporal frequency (Figure 4B). Many units showed maximal response to frequencies <2 Hz, indicative of low-pass tuning, whereas others had larger responses to higher frequencies (Figure S3). Across the population, the median preferred frequency was ~2 Hz in both genotypes, although *ReaChR rd1* units were biased toward higher frequencies than WT (U = 2.31, p = 0.02). Preference across this 1–8 Hz range was broadly retained in the dLGN (Figures 4C and 4D), with median preferred frequency at ~2 Hz for both genotypes, although units in *ReaChR rd1* retina tended to be biased toward slightly lower frequencies compared with WT (U = −5.84, p < 0.001).

In our dLGN recordings, we extended our exploration of temporal resolution to higher frequencies, presenting ~1 s blocks of sinusoidal modulations (97% Michelson contrast) between 8–30 Hz and calculating power for single unit firing at each stimulus frequency using fast-Fourier transform. At a population level, for WT, power decreased as stimulus frequency increased (Figure 4E). However, for *ReaChR rd1*, power peaked around 20 Hz. When we calculated the frequency that produced peak power across this range for individual units, we found most units had weak responses to higher frequencies, with a peak response at 8 Hz. However, a subpopulation of *ReaChR rd1* units (69/344, 20%) had a strong preference for higher temporal frequencies (19–23 Hz; Figure 4F). Units with peak power at 19–23 Hz were also present in the WT dLGN but made up a smaller proportion of the total population (37/398, 9%) and generally had smaller power values compared with *ReaChR rd1* (Figure S4). In summary, these data indicate that the *ReaChR rd1* visual system retains the low-frequency bias of WT but with an anomalous increase in responsiveness around 20 Hz in a small percentage of *ReaChR rd1* units.

### Spatial patterns

We then examined responses to spatial patterns. RGC spatial receptive fields, mapped using a sparse binary noise stimulus^27^ (Figure 5A), were comparable in *ReaChR rd1* and WT (median diameter = 255.3 and 265.8 µm, respectively, U = −1.14, p = 0.256; Figure 5B). Moving bar stimuli (Figure 5C) revealed few units with notable direction selectivity in the two genotypes (Figure 5D; median direction bias = 0.08, range = 0–0.41 for *ReaChR rd1*; median = 0.09, range = 0-0.47 for WT). However, the capacity for direction selectivity in the *ReaChR rd1* retina was confirmed by the presence of a small number of units with relatively high direction-selective indices (Figure 5E). Given that such strongly selective units were relatively rare in both genotypes, it was not possible to determine whether their frequency differed between *ReaChR rd1* and WT.

### Response reproducibility

**Figure 6:**
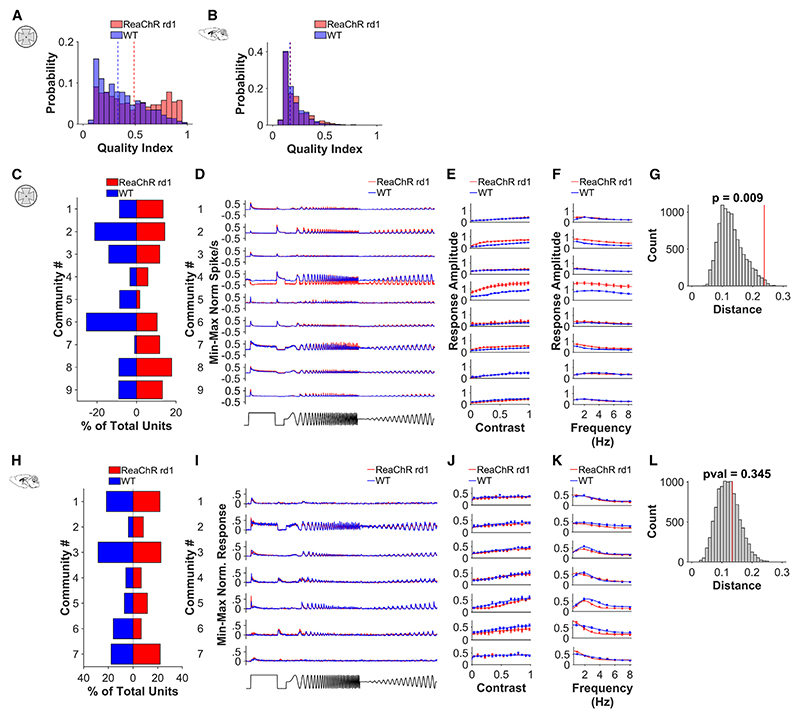
Community detection (A and B) Histogram of a quality index for PSTH data (100 ms bins) for (A) retina (*ReaChRrd1* n = 741, WT n = 1,005) and (B) dLGN (*ReaChRrd1* n = 344, WT n =398) units. (C and H) Distribution of units from each genotype across different communities in (C) retina (*ReaChR rd1* n = 741, WT n = 741) and (H) dLGN (*ReaChR rd1* n = 344, WT n = 344). (D and I) Mean PSTH (25 ms bins) for units with each community for each genotype in (D) retina and (I) dLGN. (E and J) Contrast sensitivity function fit to mean response to contrast chirp for (E) retina and (J) dLGN. (F and K) Temporal tuning function fit to mean response to temporal chirp for (F) retina and (K) dLGN. (G and L) Comparison of the distribution of retinal units across communities. We calculated the “distance” (Euclidian norm of the difference in the relative proportion of neurons in each community) between *ReaChR rd1* and WT (shown by the red line) and compared this with a null distribution obtained by randomly shuffling replicates between two groups for (G) retina and (L) dLGN. The resulting p value is shown above each figure panel. Error bars show SEM. See also Figures S5 and S6.

Having defined some fundamental visual capabilities of *ReaChR rd1*, we turned our attention to visual response reliability. To this end, we used the quality index^22^ as a measure of reproducibility for firing patterns across repeated presentations of the chirp stimulus (1 = identical every trial). There was no suggestion that retina or dLGN responses were less reproducible following retinal degeneration. In fact, in the retina, the quality index tended to be higher in *ReaChR rd1* than in WT (Figure 6A; median = 0.49 for *ReaChR rd1*, 0.33 for WT, U = 9.94, p < 0.001). Overall, measures of the quality index were lower in the dLGN and the difference between genotypes was no longer apparent (median = 0.17 for *ReaChR rd1*, 0.17 for WT, U = 0.07, p = 0.944; Figure 6B).

### Response diversity

A key characteristic of the visual system is diversity in feature selectivity. Differences in polarity and persistence of responses to the light step (Figure 2) confirm this diversity is retained to some extent in *ReaChR rd1*. Similarly, across both dLGN and retina *ReaChR rd1* units, we found considerable variability in responses to both contrast and temporal chirp. We finally set out to capture the multi-dimensional nature of this response diversity for comparison between genotypes by combining probabilistic clustering and community detection.

First, sparse principal components based on pooled WT and *ReaChR rd1* responses to chirp stimulus were grouped using probabilistic clustering.^22,31^ Across 50 runs of this method, we found between 19 and 25 clusters in the retina (from n = 741 randomly sampled WT units from 11 mice and n = 741 *ReaChR rd1* units from 5 mice) and between 8 and 13 clusters in the dLGN (from n = 344 *ReaChR rd1* units from 5 mice and n = 344 randomly sampled WT units from 4 mice). For most runs, individual clusters contained units from both genotypes across retina (27/50 runs) and dLGN (48/50 runs) data (Figure S5). Indeed, across all runs, none produced a cluster containing only WT units. This suggests that the diversity of the visual response to the chirp stimulus is well preserved in *ReaChR rd1* animals.

To facilitate more detailed comparisons of the distribution of WT and *ReaChR rd1* units across clusters, we employed a downstream community detection analysis to obtain repeatable clustering results. In brief, a similarity matrix was constructed from the multiple runs of probabilistic clustering, and community detection^32,33^ applied to group units into functional output channels (referred to as communities). The community detection method reduced variability in the number of groups produced and in the sets of units associated with those groups (Figure S6).

Application of community detection returned 9 communities of retinal responses (Figures 6C–6F). Notably, all communities contained units from both genotypes indicating that no category of response was entirely absent in *ReaChR rd1*. However, there was a statistically significant difference in the overall distribution of units across communities between WT and *ReaChR rd1* retinas (Figure 6G; p = 0.01, see STAR Methods for statistical test details). Accordingly, some communities were clearly dominated by units from one genotype. For example, communities 5 (p = 0.027, 84% WT units) and 6 (p = 0.004, 71% WT units) with transient ON-OFF responses were both found more often in WT animals and may be considered underrepresented in *ReaChR rd1*. In comparison, we found that community 7, characterized by sustained ON responses (Figures 6D–6F), included significantly more *ReaChR rd1* units (p = 0.001, 93% *ReaChR rd1*). The reduction in the number of biphasic units, as well as the corresponding increase in ON units, in the *ReaChR rd1* retina was consistent with our previous analysis of response categories (Figure 2).

In the dLGN, 7 communities were detected (Figures 6H–6K). The community encompassing OFF responses (community 6) was the most biased in favor of WT (70% WT units). However, in the dLGN, there was no statistically significant difference in distribution across the categories (Figure 6L; p = 0.345). Overall, our data reveal a remarkable consistency in the categories of response to the full-field stimuli between *ReaChR rd1* and WT.

## Discussion

Our data reveal that much of the signal transmission and processing capacity of the intact visual system is retained in advanced retinal degeneration. Thus, many of the properties of the WT visual code at the level of RGCs and the dLGN can be recreated using ReaChR expressed in OBCs in the *rd1* retina. *ReaChR rd1* units showed spatial receptive fields of equivalent size as in WT and retained the capacity for direction selectivity. In terms of fundamental stimulus-response characteristics, much of the diversity responsible for the rich visual code in WT mice was retained in *ReaChR rd1* animals. At the level of the dLGN and retina, *ReaChR rd1* units could be sustained or transient; ON, OFF, or biphasic; high or low contrast sensitivity; and band- vs. long-pass temporal frequency tuning. Moreover, both WT and *ReaChR rd1* units were found in all communities based on stimulus-response properties, indicating that the diversity of functional output channels is at least partly conserved in advanced retinal degeneration.

Those features of the WT visual code preserved in the *ReaChR rd1* mice must be robust both to the secondary degeneration and anatomical reorganization described for *rd1* mice^34–38^ and to the loss of signal processing that normally occurs upstream of OBCs. Literature on the degenerate retina’s functional capacity is limited, mostly focusing on changes in photoreceptor-driven responses at earlier stages of degeneration. Although contrast sensitivity seems well preserved in those studies,^39,40^ some loss of function has been found, including attenuated response amplitude, longer onset latency, smaller receptive fields, and the loss of inhibitory/OFF responses in *rd1* mice.^40^ Our data are consistent with the view that these features are more a consequence of photoreceptor depletion/dysfunction than network reorganization.

In terms of pre-OBC processing, the opportunities for signal processing absent in the *ReaChR rd1* retina include photoreceptor adaptation, rod and cone gap junction communication, horizontal cell networks and feedback to photoreceptors, and signal sculpting at the photoreceptor to bipolar cell synapse. The fact that many aspects of visual signal processing can occur without these elements is encouraging for the prospects for vision restoration and highlights the capacity of the inner retinal circuitry involving bipolar, amacrine, and ganglion cells. Of particular significance is the ability of *ReaChR rd1* retinal units to code contrast across a range of background irradiances. Although less efficient than in WT, *ReaChR rd1* units were clearly encoding relative, rather than absolute, light intensity. In intact retinas, the sensitivity normalization required for contrast coding can arise in photoreceptors thanks to photopigment bleaching and phototransduction cascade adaptation, but also encompasses post-receptoral mechanisms.^41–44^ The presence of this behavior in the *rd1* retina suggests that sensitivity normalization in downstream circuitry is intact and is sufficient for contrast coding across different backgrounds. In a therapeutic context, this capacity could help to normalize responses in the face of quantitative variations in photopigment expression (and thus effective photon flux) across the target cell population produced by imperfect gene delivery.

An important feature of the *ReaChR rd1* dataset is that unit-to-unit diversity in stimulus-response characteristics is retained. Critical differentiators of visual feature selectivity in WT (including contrast sensitivity, temporal frequency preference, response polarity, and sustainedness) were captured in the *ReaChR rd1* dLGN and retina responses and varied across the population. Crucially, not only was this variability retained by *ReaChR rd1* but it also produced the same functional output channels in both genotypes as revealed by community detection. Thus, we found evidence that although the relative frequency of units with particular response properties may differ in *ReaChR rd1* mice, no functional category was entirely absent from the *rd1* visual system. Although this observation is encouraging, it does not follow that the rd1 visual code is entirely “normal.” A wider array of stimuli and test parameters would be required to assay the full range of visual channels described by others.^22,23,25^ Moreover, future work will be required to determine whether given stimulus-response characteristics are a feature of neurons of the same neuroanatomical classes in degenerate and WT animals. Nevertheless, our results do suggest that the ability to support a great diversity of visual properties is retained following advanced retinal degeneration.

The description of *ReaChR rd1* vision in this study necessarily draws from a constrained range of visual stimuli. Given the limited sensitivity range of *ReaChR* responses, these animals will certainly lack richness in the WT visual code arising from irradiance-dependent changes in network function or pre-OBC processing and may also be deficient in other categories of visual signal processing not probed by our stimuli. Even within our stimulus set, there were some key differences with WT. Some can likely be attributed to the optogenetic actuator, rather than deficiencies in visual circuit performance, per se. For example, we consistently found that *ReaChR rd1* retinas only responded at higher irradiances. These results undoubtedly reflect the reduced photosensitivity of *ReaChR*^11^ in OBCs compared with native rods and cones whose high photopigment density and signal-amplifying phototransduction cascades increase sensitivity.^45–47^ Similarly, the shorter response onset latency seen in *ReaChR rd1* mice may be caused by rapid *ReaChR* temporal kinetics,^28^ but it could also be due to target cell type. By expressing *ReaChR* in *rd1* second-order neurons (OBCs), the retinal circuit is one synapse shorter, which could also decrease latency measured in RGCs and dLGN.

Target cell type is also the most parsimonious explanation for the shifts in the distribution of response types. In the dLGN and retina, the proportion of ON responses increased (particularly sustained ON based on community detection), whereas OFF/biphasic responses decreased in *ReaChR rd1*. In the WT retina, light stimulation leads to activation of both rod and cone OBCs, along with OFF bipolar cells.^48–50^ However, light stimulation of the *ReaChR rd1* retina likely results only in activation of OBCs. Although rod bipolar cells connect to OFF pathways via AII amacrine cells,^51–53^ the absence of direct OFF bipolar cell activation in *ReaChR rd1* retinas may explain the reduced frequency of OFF responses compared with WT.

Several differences observed in *ReaChR rd1* compared with WT were harder to categorize, including the larger response amplitude in the retina, the dLGN subpopulation with a preference for 19–23 Hz, and the highly reproducible responses in the retina. The optogenetic tool or *Grm6*^*Cre*^ transgenic model could be a factor, but subtle changes from retinal remodeling after degeneration could also contribute. In the case of increasingly uniform responses seen in *ReaChR rd1* retinas, it is unclear if this is necessarily positive or negative.^41–43^ Under certain conditions, noise can be important for information handling in neuronal circuits,^54^ and losing this may result in the less naturalistic encoding of visual information. But equally, improved signal to noise could be advantageous from a vision restoration perspective.

Overall, our findings are encouraging for regenerative medicine in the central nervous system in general and vision restoration in particular. OBC targeting in the degenerate retina appears to have the potential to restore diverse and complex visual responses, resulting in a visual code approximating that of WT animals. This implies the functional capacity of deafferented circuits can be substantially retained. In the case of vision restoration, our results raise the hope that, provided efficient transgene delivery can be achieved, optogenetic therapies could restore a high degree of visual function.

## Star⋆Methods

Detailed methods are provided in the online version of this paper and include the following:

- KEY RESOURCES TABLE
- RESOURCE AVAILABILITY
  ○ Lead contact
  ○ Materials availability
  ○ Data and code availability
- EXPERIMENTAL MODEL AND SUBJECT DETAILS
  ○ Animals
  ○ Ethical statement
- METHOD DETAILS
  ○ Transgenic mouse line
  ○ Immunohistochemistry
  ○ Retinal MEA electrophysiology
  ○ In vivo electrophysiology
  ○ Visual stimuli paradigms
- QUANTIFICATION AND STATISTICAL ANALYSIS
  ○ Analysis of step response
  ○ Analysis of contrast sensitivity
  ○ Analysis of temporal frequency tuning
  ○ Receptive Field mapping and Direction Selectivity
  ○ Clustering and community detection

## Star⋆Methods

### Key Resources Table

**Table:** 

REAGENT or RESOURCE	SOURCE	IDENTIFIER
Antibodies		
Chicken polyclonal anti-GFP	AVES labs	Cat# GFP-1020; RRID: AB_10000240
Rabbit monoclonal anti-PKCalpha	Abcam	Cat# ab32376; RRID: AB_777294
Experimental models: Organisms/strains		
C56Bl/6 mice	Envigo & University of Manchester	RRID: MGI:5811150
*ReaChR-mCitrine* mice	Jax Laboratories	RRID: IMSR_JAX:026294
*Grm6Cre* mice	Robert Duvoisin (Oregon Health & Science University)	RRID: MGI: 4411993
*Pde6b*^*rd1*^ mice	University of Manchester	RRID: MGI: 1856373
Software and algorithms		
Matlab R2020a	Mathworks	RRID: SCR_001622
sPASM toolbox	Sjöstrand et al.^55^	http://www2.imm.dtu.dk/projects/spasm/
Brain Connectivity toolbox	Rubinov and Sporns^33^	RRID: SCR_004841

### Resource Availability

#### Lead contact

Further information and requests for resources and reagents should be directed to and will be fulfilled by the lead contact, Mark Hankins (mark.hankins@eye.ox.ac.uk)

#### Materials availability

There are restrictions on the availability of the *Grm6Cre; ReaChR; Pde6b*^*rd1*^ transgenic mouse line due to a material transfer agreement for the *Grm6Cre* mouse line.

### Experimental Model and Subject Details

#### Animals

Retinal MEA recordings were conducted on 5 *ReaChR Grm6*^*Cre/WT*^ *Pde6b*^*rd1/rd1*^ mice (4 females, 1 male at 144-169 days old), 3 *ReaChR Grm6*^*WT/WT*^ *Pde6b*^*rd1/WT*^ mice (2 females, 1 male at 144-158 days old) and 8 C57Bl/6 mice (Envigo, 6 females, 2 males at 150-164 days old). *In vivo* dLGN recordings were conducted on 5 *ReaChR Grm6*^*Cre/WT*^ *Pde6b*^*rd1/rd1*^ (1 female, 4 males, 153-166 days old) and 4 C57Bl/6 mice (University of Manchester, 3 females, 1 male, 141-196 days old). *ReaChR Grm6 Pde6b*^*rd1*^ mice were generated by University of Oxford. Animals were group housed, provided with food and water ad libitum throughout their life and kept on a 12:12 light dark cycle.

#### Ethical statement

Experiments were conducted in accordance with the Animals, Scientific Procedures Act of 1986 (United Kingdom) and approved by the University of Manchester and University of Oxford ethical review committees.

### Method Details

#### Transgenic mouse line

A new strain – *Grm6Cre; ReaChR; Pde6b*^*rd1*^ – was created for this study by breeding *Grm6*^*Cre/WT* 29^ (MGI:4411993, kindly shared by Robert Duvoisin, Oregon Health and Science University); *Pde6b*^*rd1/rd1*^ mice maintained at the University of Manchester, with *ReaChR-mCitrine* mice^56^ (MGI: 5605725) obtained from Jax laboratories (strain 026294). Mice were bred to be homozygous for *ReaChR-mCitrine*, heterozygous for *Grm6 Cre* and homozygous for *Pde6b rd1* and are on a mixed C57Bl/6 x C3H background. These mice express *ReaChR-mCitrine* primarily in ON bipolar cells (Figure S1). By 5 months old, *rd1* mice are in later stages of degeneration, following loss of rod photoreceptors and secondary cone degeneration. Prior to experiments, *ReaChR rd1* mice were kept in lighting conditions below threshold for activating *ReaChR*.^11,28,56^ Genotyping was performed using Immomix mix red (Bioline) for Cre (Fwd 5’-TCAGC AGGTTGGAGACTTTC, Rev 5’-TTCACAACCTGTCAGACCAC, 800bp band) *rd1* (Fwd 5’-TGACAA TTACTCCTTTTCCCTCAGTCT, WT Rev 5’-GTAAACAGCAAGAGGCTTTATTG GGAA, *rd1* Rev 5’ – GCATTAATTCTGGGGCGCATG. WT 400bp, *rd1* 550bp band) and *ReaChR* (Fwd 5’ - CTTCCCTCGTGATCTGCAA, WT Rev 5’ – CAGGACAACGCCCACACA, ReaChR rev 5’ – GTTATGTAACGCGGA ACTCCA. WT 96bp band, *ReaChR* 140bp band) or using an automated genotyping service (Transnetyx). For retina studies, recordings from wildtype C57Bl/6 mice were supplemented with data from *ReaChR; Grm6*^*WT/WT*^; *Pde6b*^*rd1/WT*^ which are visually intact and do not express *ReaChR-mCitrine* or Cre recombinase in the retina.

#### Immunohistochemistry

Immunostaining of retina sections was performed as described previously.^57,58^ Visualization of *ReaChR-mCitrine* was enhanced using a chicken polyclonal anti-GFP antibody diluted 1:1000 that also recognizes *mCitrine* (GFP-1020, AVES labs). Anti-PKCa antibody diluted 1:1000 (ab32376, Abcam) was used to label rod ON-bipolar cells. Fluorescence images were collected using an inverted LSM 710 laser scanning confocal microscope (Zeiss) and Zen 2009 image acquisition software (Zeiss). Individual channels were collected sequentially. Laser lines for excitation were 405nm, 488nm and 561nm, with emissions collected between 440-480, 505-550 and 580-625nm for blue, green and red fluorescence respectively. Images were collected using a x40 objective with images collected every 1µm in the z-axis. Global enhancement of brightness and contrast was performed using ZenLite 2011 software (Zeiss).

#### Retinal MEA electrophysiology

Following enucleation, retinas were dissected under dim red light (>610 µm) and transferred to glass-bottomed MEA chambers (Multi Channel Systems, Reutlingen, Germany), with ganglion cell side facing down. MEA chambers (containing 252 electrodes, each 30mm in diameter and spaced 100 µm apart) were placed into the MEA recording device (MEA2100-256 system; Multi Channel Systems) and positioned within the light path of an inverted Olympus IX71 microscope. Retinae were perfused with Ames’ media bubbled with 95% O_2_ /5% CO_2_ (pH 7.3) and maintained at 34 °C. Recorded signals were collected, amplified, and digitized at 25 kHz using MCS Experimenter Software (Multi Channel Systems). Retinae were perfused for at least 30 min in darkness before commencement of experiments.

A white LED light source with a daylight spectrum (Thor labs, SOLIS-3C) and an arbitrary waveform generator (RS components, RSDG2000X Series) were used to generate ‘chirp’ light stimuli.^22^ Intensity of light stimuli was controlled via motorised filter wheels containing neutral density filters (0-6 log units, Thor Labs). Sparse spatial noise and moving bar light stimuli were generated as described previously.^27^ Briefly, a narrowband 565nm OptoLED light source (Cairn Research, Faversham, UK) and motorized filter wheels containing neutral density filters (ThorLabs, Newton, USA) were used in combination with a digital mirror “pattern stimulator” device (Polygon400, Mightex Systems, Toronto, Canada) to create sequences of defined light patterns. The power of all light stimuli (in microwatts/cm^2^) were measured at the sample focal plane using an in-line power meter (PM160T; ThorLabs), and units converted to photons per cm^2^/s using an irradiance toolbox^59^ from http://www.eye.ox.ac.uk/team/principal-investigators/stuart-peirson. All devices were automatically controlled and synchronized by a Digidata 1440A digital I/O board (Axon Instruments, Molecular Devices, San Jose, USA) and a PC running WinWCP software (J Dempster, Strathclyde University, Glasgow, UK).

Spike sorting of retina MEA data was performed using SpikeSorter software^60^ (Version 4.77b Nicholas Swindale, UBC). Raw data was filtered using a high pass 4-pole 500Hz Butterworth filter. Event detection was based on 4-5x median noise signal, with window width of 0.24ms. Results of automatic spike sorting were manually inspected and corrected using SpikeSorter software and Offline Sorter (Plexon) before being exported to Matlab for further analysis.

#### In vivo electrophysiology

As described previously,^61^ mice were anaesthetized by intraperitoneal injection of urethane (1.4-1.5 g/kg, Sigma-Aldrich) and placed in a stereotaxic frame. The surface of the skull was exposed and a small hole was drilled 2.2mm lateral and 2.2mm posterior to bregma. The pupil of the eye contralateral to the craniotomy was dilated using atropine (1% in saline, Sigma-Aldrich) and moistened using a small amount of mineral oil or Lubrithal (Dechra) to prevent corneal dryness. Multi-electrode arrays (A4×16-Poly2-5mm-23s-200-177-A64, NeuroNexus), consisting of 4 shanks (200µm apart) with 16 recording sites per shank, were used. Shanks were coated in CM-DiI (Fisher Scientific), positioned at 2.2mm lateral and 2.2mm posterior to bregma and inserted to a depth of 2.5-3mm targeting the dorsal lateral geniculate nucleus.^30^ Successful recording from dLGN was confirmed by presence of light responsive units in response to 5s step of white light (5s interstimulus interval, 10 trials, 15log and 16log photons/cm^2^/s for WT and *ReaChR rd1* respectively). Once light responsive units were identified, mice were left for 30mins to dark adapt and allow neural activity to stabilise. Neural signals were acquired using a Recorder64 system (Plexon), and were amplified (x3000), high-pass filtered at 300Hz, digitised at 40kHz and stored continuously in a 16bit format.

Multiunit activity was saved and analysed offline using Offline Sorter (Plexon). In a few cases, once recording was complete, the recording probes were raised and moved ~0.2mm posterior or anterior before insertion at an additional site, to sample a non-overlapping region of the dLGN. Once dLGN experiment was complete, mice were killed by cervical dislocation, brains removed and fixed in 4% paraformaldehyde. Single unit activity was isolated using an automated template-matching based algorithm Kilosort^62^ and exported as ‘virtual tetrodes’ (spike waveforms detected across 4 adjacent channels) to be manually checked in Offline Sorter (Plexon). Single unit isolation was confirmed as described in Mouland et al.^61^

A CoolLED pE-4000 light source connected to a liquid light guide fitted with a diffuser (Edmund Optics) was used to deliver full-field stimuli, positioned at a distance of approximately 5mm from eye contralateral to recording site. White light was used for all stimuli, consisting of output from 4 LEDs with peak emission at 385nm, 470nm, 550nm, 660nm. Irradiance was controlled using neutral density filters to produce a range from 12-16 log effective photons/cm^2^/s.

#### Visual stimuli paradigms

For both retina and dLGN, responses were recorded to a standardised set of full-field stimuli.^22^ This consisted of a 3s step from dark to maximum light intensity, followed by 2s of dark, 2s at half maximum light intensity, an 8s temporal chirp (sinusoidal modulation between dark and maximum intensity at 1-8Hz accelerating at rate of 1Hz/s), 2s at half maximum light intensity, an 8s contrast chirp (sinusoidal modulation at 2Hz increasing from 3% to 97% contrast), 2s at half maximum intensity and finally, 3s of dark.

Due to difference in light sensitivity between WT and *ReaChR rd1*, a different range of intensities was tested for each group. For *in vivo* electrophysiology this was between 13.96-16.97 log photons/cm^2^/s for *ReaChR rd1*, while for WT, this was 12.99-16.97 log photons/cm2/s, equivalent to 5674 to 3.8 x 10^7^ rhodopsin photoisomerisations per rod per s, 6384 – 4.8 x 10^7^ M-opsin photoisomerisations per cone per s and 1095 – 1.34 x 10^7^ S-opsin photoisomerisations per cone per s. For retinal recordings, this was 14.9-17.4 log photons cm^2^/s for *ReaChR rd1*. For WT, this was 11.9-16.9 log photons cm^2^/s, equivalent to 2735 – 2.74 x 10^8^ rhodopsin photoisomerisations per rod per s, 3566 – 3.57 x 10^8^ M-opsin photoisomerisations per cone per s and 33.9 – 3.40 x 10^6^ S-opsin photoisomerisations per cone per s.

Photoisomerisations per rod, M cone or S cone per second were calculated as in Tikidji-Hamburyan et al.,^63^ with spectrum converted to photons/cm^2^/s/nm, convolved with the normalised opsin sensitivity spectrum, and multiplied by the effective collection area^64^ of rods (0.85µm^2^) and cones (1mµ^2^). For *in vivo* electrophysiology, the light spectra was corrected for lens transmission and retinal irradiance calculated before conversion to photoisomerisations per s.

For dLGN, responses were also recorded to higher frequency sinusoidal modulations between dark and maximum light intensity. From dark, we presented consecutive blocks of 1s of modulation at 7.8, 11.4, 15.9, 19.1, 22.7, 26.4 and 30.4Hz, followed by 3s of dark. These were conducted over same intensity range as full-field stimuli.

Receptive fields were mapped using a sparse binary noise stimulus, applied as a 16 × 16-chessboard pattern with a 48.8mm-wide pixel width (256 consecutive frames), with a frame duration of 1s. Light intensity at bright fields was either 14.2 (for WT) or 16.2 (for *ReaChR rd1*) log photons cm^2^/s, 565nm LED, contrast between bright and dark fields was 1:1000.

Direction selectivity was assessed using a sequential projection of moving wide bars into four distinct directions (0, 90, 180 and 270 degrees) at 200um/s. Light intensity was either 14.2 (for WT) or 16.2 (for *ReaChR rd1*) log photons cm^2^/s, 565nm LED, with a contrast of 1:1000. The bar moves at a rate of 500µm/s (total time 4s) and was the same width as the entire visual display.

### Quantification and Statistical Analysis

Unless otherwise specified, graphs show mean with error bars showing standard error of the mean, sample size is given in figure legends and refers to number of retinal and dLGN units (details are described in methods) and Mann-Whitney U-tests were used to compare between genotypes, with significance determined as p < 0.05.

#### Analysis of step response

Peri-event spike histograms (PSTH) were generated with bin size of 25ms. Error bars for PSTH and other graphs show standard error of the mean, unless otherwise specified. Light responsive units were identified using confidence limits test based on responses to the 3s step of the chirp stimulus: units were classified as significant if spike firing rate during the response window was greater than 2 standard deviations above (activation) or below (suppression) mean firing rate during baseline window – equivalent to 95% confidence limit. We used 3 different confidence limit (CL) tests; activity during the first 2s of the chirp step compared with 1s before step onset (transient ON), activity during 0.5s after step offset compared with last 1s of step stimulus (OFF) and activity during last 1s of step stimulus compared with 1s before step onset (sustained ON).

We classified light responsive units into 4 different categories based on outcome of these confidence limits tests: 1) Transient ON units were significant for transient ON test, with no sig. excitation for sustained ON and no sig. response for OFF test, 2) Sustained ON units were sig. for transient and sustained ON tests with no sig. activation for OFF test. 3) Biphasic units were sig. activation for transient ON and OFF tests, with no sig. response to sustained ON test. 4) OFF/suppressed ON units had significant suppression for transient and sustained ON tests and had significant responses with OFF test. Units with a quality index below 0.1,^22^ a measure of response variability between repeated trials, were excluded from further analysis. Firing rate was then normalised to baseline by subtracting the average activity during 2s before step onset.

Irradiance response curves were constructed based on maximum normalised firing rate during 3s step, by fitting Hill slope curve with 4 best-fit parameters (Top, Bottom, logEC50 and Slope) identified using non-linear regression. Irradiances which produced responses closest to 75% maximum were identified and used for comparisons between WT and *ReaChR rd1* groups. The 75% threshold was used as it would provide sub-saturating responses in the majority of units, while ensuring good signal to noise ratio and as large a sample size of responsive units as possible.

Equation for Hill slope curve is

$$\text{Re}sponse\;=\frac{\;Bottom\;+(\;Top\;-\;Bottom\;)}{1+10^{(LogEC50\;-\;LogPhotons\;)*\;Slope\;}}$$

where LogPhotons is intensity in log photons/cm^2^/s

ON-OFF Bias index and Transience index were calculated using previously described methods.^20,27^ Latency for onset of 3s step was defined as timing of first bin where response was outside the confidence limits for the transient ON confidence limit test. Light responsive units that did not respond to onset of 3s step (ie: OFF units) were excluded from this analysis.

### Analysis of contrast sensitivity

The response amplitude (maximum – minimum normalised firing rate during each period of the contrast chirp stimulus) was normalised to baseline activity during 1s before contrast chirp onset for each unit. This was plotted against Michaelson contrast and then fit using a contrast sensitivity function, or Naka-Rushton curve,^65^ using least-squares minimisation to identify 4 best-fit parameters (top, bottom, C50 and slope).

Equation for Naka-Ruston curve is

$$\;\;\text{Re}sponse=\;\;Bottom\;+\left(Top*\frac{C^{n}}{C^{n}+C50^{n}}\right)$$

Where *n* = slope, *C* = Michelson contrast and C50 is contrast that produces half maximum response. C50 was constrained between 0 and 1, and slope was constrained between 0 and 10. Only curve fits with R^2^ > 0.5 and spiking in >10% of bins were used for comparison of contrast sensitivity parameters.

For analysis of contrast coding, the change in irradiance between peak and trough of chirp modulation for each period of contrast chirp stimulus (Δ Irradiance). Response amplitude for contrast chirp (calculated as above) at each background irradiance was then plotted against Michelson contrast or Δ Irradiance for light responsive units. Mean contract response amplitudes were then fit with contrast sensitivity function (for amplitude vs contrast) or Hill-slope curve (for amplitude vs Δ Irradiance).

For analysis of contrast coding at an individual level, we first identified light responsive units that had contrast sensitivity functions with R^2^ > 0.5 for each background irradiance. We then calculated the correlation between C50 from contrast sensitivity curve and background irradiance, as well as the slope of the linear trendline.

### Analysis of temporal frequency tuning

For analysis of temporal chirp, mean response amplitude (calculated as above) for each temporal frequency was fitted with a half-Gaussian model^66^ using least-squares minimisation to identify 5 best-fit parameters (low baseline, high baseline, Gaussian spread, peak response and peak frequency).

Equation for Half Gaussians is:

Response=b1+(a−b1)*e−[p−ws]^2forw<p

Response=b2+(a−b2)*e−[p−ws]^2forw>p

Where *w* is the temporal frequency (Hz), *p* is the temporal frequency (TF) that produces peak response, *a* is the maximum response amplitude at optimum TF, *s* is the Gaussian spread, *b1* is the baseline for frequencies lower than peak TF, *b2* is the baseline for frequencies greater than the peak TF. Only curve fits with R^2^ > 0.5, spiking in > 10% of bins and Gaussian spread > 0.51 were used for comparison of temporal frequency tuning parameters. Peak temporal frequency was rounded to nearest integer to address the limited resolution of temporal frequency analysis (sample every 1Hz).

For analysis of faster frequencies, for each stimulus frequency, we generated a PSTH with data in 10ms bins and performed a fast-Fourier transform. We then extracted the mean power for the 3 frequencies closest to stimulus frequency and plotted this as a function of stimulus frequency.

### Receptive Field mapping and Direction Selectivity

Receptive field diameter was calculated using the method described in Lindner et al.^27^ with minor modifications: For each stimulus frame, PSTHs were calculated for an interval starting 500ms before the beginning of frame, to 500ms after the end of frame (bin size 250 ms). The absolute peak difference in spike firing rate over each PSTH was then calculated, which enabled analysis of ON, OFF, ON-OFF and sustained units likewise. Resulting values were multiplied with a 2D binary matrix representing the stimulus pattern during that particular frame. Thereby, observed responses could be matched to individual stimulus locations. Resulting matrices were then collapsed and normalized. Receptive field centre positions were detected, and the width of the receptive field was determined by Gaussian fits. Receptive field centre positions were detected on smoothed data to minimize the effect of possible noise. Receptive fields which had their calculated maximum at the edge of the field of view were excluded from analysis.

The Direction Selectivity Index was calculated as described by Farrow and Masland^20^: For each responsive units, spike firing rate during the presentation of the moving bar was computed for each of the four directions of movement independently. A direction selectivity vector for each responsive unit was computed. The length of the vector was normalized resulting in the Direction Selectivity Index. This method of calculating direction selectivity was used in order to detect units with preference for diagonal motion, despite only having two axes of stimulus motion (horizontal and vertical).

For spatial stimuli, units were identified as light responsive based on response to a 1s flash. Units were classified as light responsive if there was a change in spike firing rate of >10 spikes/s and a relative change in number of spikes > 20% during flash compared to baseline.

### Clustering and community detection

To assess reproducibility across trials, the quality index^22^ was calculated for PSTH data in 100ms bins.

Sparse Principal components were generated for the following windows of chirp stimulus – Step (0.5-4.5s), temporal chirp (6.5 – 15.5s) and contrast chirp (17.5-24.5s) – using the SPaSM toolbox,^55^ as described in Caval-Holme et al.^31^ and Baden et al.^22^ This allows the extraction of response features that are localised in time. To avoid bias due to smaller number of light responsive units in *ReaChR rd1* compared to WT datasets, we randomly sampled a subset of light responsive units from WT datasets to obtain same sample size between genotypes. We pooled mean PSTH (25ms bins) for these light responsive units from both groups and extracted up to 31 features with 5 non-zero time bins. We then discarded those that accounted for < 1% of the variance. Response features that met these criteria for each window were then combined to produce a total of 16 features for retina data and 31 features for dLGN.

sPCs from pooled *ReaChR* and WT data were then clustered with a mixture of Gaussian models, a probabilistic model using random initialisation. According to this method, the optimum number of clusters is determined based on the lowest Bayesian information criteria, which rewards fit but penalises complexity, and a Bayes factor below 6, as used by Caval-Holme et al.,^31^ as a threshold for when there was no longer evidence for further splitting.

The probabilistic clustering was repeated 50 times. A pairwise similarity matrix was generated based on how frequently each unit pair clustered together (with 0 being never clustered together and 1 being always clustered together). Based on this similarity matrix, a community detection algorithm^32^ using the Brain connectivity toolbox (Rubinov and Sporns^33^ at https://sites.google.com/site/bctnet/) was used to group units into functional output channels.

To compare distribution of units across communities between two groups, we calculated the distance (Euclidian norm of the difference in the mean relative proportion of neurons in each cluster) between *ReaChR rd1* and WT (shown by red line) and compared this with a null distribution obtained by randomly shuffling retinal recordings between two groups 10,000 times. To compare proportion of units from each group within each cluster, we first calculated the % of total units from each group in a given community for each recording, and we used these averages to calculate the % difference between *ReaChR rd1* and WT. This % difference was compared with a null distribution generated as above.

## Supplementary Material

Supplementary Information

## Data Availability

All data reported in this paper will be shared by the lead contact upon request. This paper does not report original code. Any additional information required to reanalyze the data reported in this paper is available from the lead contact upon request.

## Highlights

- ReaChR in bipolar cells restores high-quality visual responses in *rd1* mice
- *ReaChR rd1* stimulus-response characteristics show equivalent diversity to WT
- *rd1* visual system can parse visual features into multiple output channels
- Remodeling after photoreceptor loss has a limited impact on *rd1* functional capacity
