# Supplementary material for: Functional integrity of visual coding following advanced photoreceptor degeneration
Source: Curr Biol. Author manuscript; Available in PMC 2026 Aug 6. (PMC7619324; doi:10.1016/j.cub.2022.12.026)
Supplement: Supplementary file 1 [file EMS216073-supplement-Supplementary_Information.pdf]

Current Biology, Volume *33*

Supplemental Information

Functional integrity of visual coding

following advanced photoreceptor degeneration

Jessica Rodgers, Steven Hughes, Moritz Lindner, Annette E. Allen, Aghileh S. Ebrahimi, Riccardo Storchi, Stuart N. Peirson, Robert J. Lucas, and Mark W. Hankins

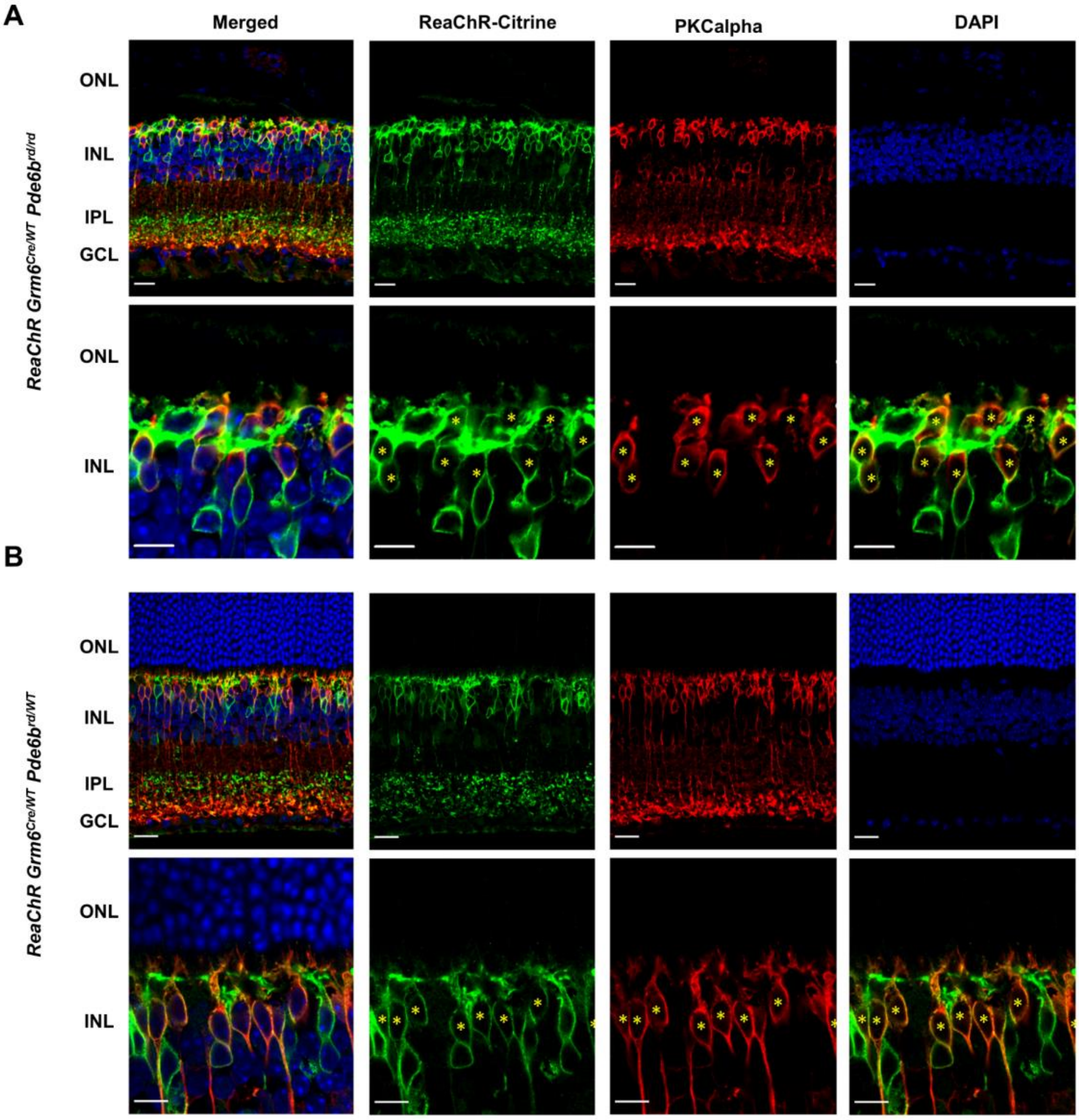

Figure S1. ReaChR expression in the degenerate and non-degenerate retina. Related to Figure 1

a-b) Retinal sections for a) retinally degenerate *ReaChR Grm6Cre rd homozygous* and b) non degenerate *ReaChR Grm6Cre* rd heterozygous mice counterstained for *ReaChR-mCitrine* (anti-GFP, green), PKCalpha (red), and DAPI (blue). Scale bar = 20µm for top row, 10µm for bottom row. * indicates PKCalpha labelled rod ON bipolar cells also labelled for *ReaChR.mCitrine*. Note that *ReaChR mCitrine* expression is also detected in non-PKCalpha labelled cells consistent with expression in cone ON bipolar cells[S1] . ONL = outer nuclear layer, INL = inner nuclear layer, IPL = inner plexiform layer, GCL = ganglion cell layer.

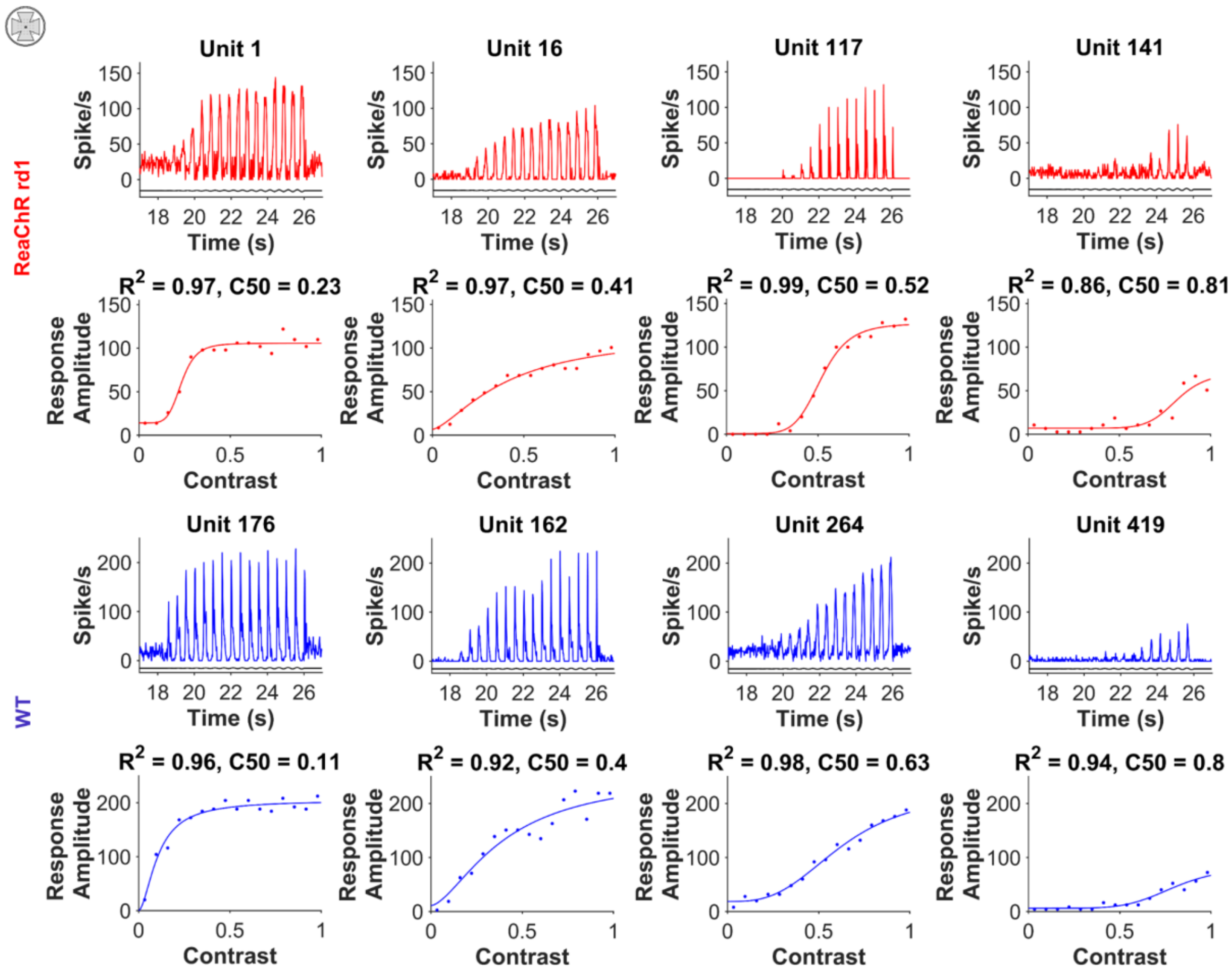

Figure S2. Diverse contrast sensitivity in *ReaChR rd1* and WT retinas
Related to Figure 3
PSTH (top) and contrast sensitivity function (bottom) for 4 example units from a) *ReaChR rd1* (red) and b) WT (blue) retinas, showing diversity in C50 and slope within each genotype.

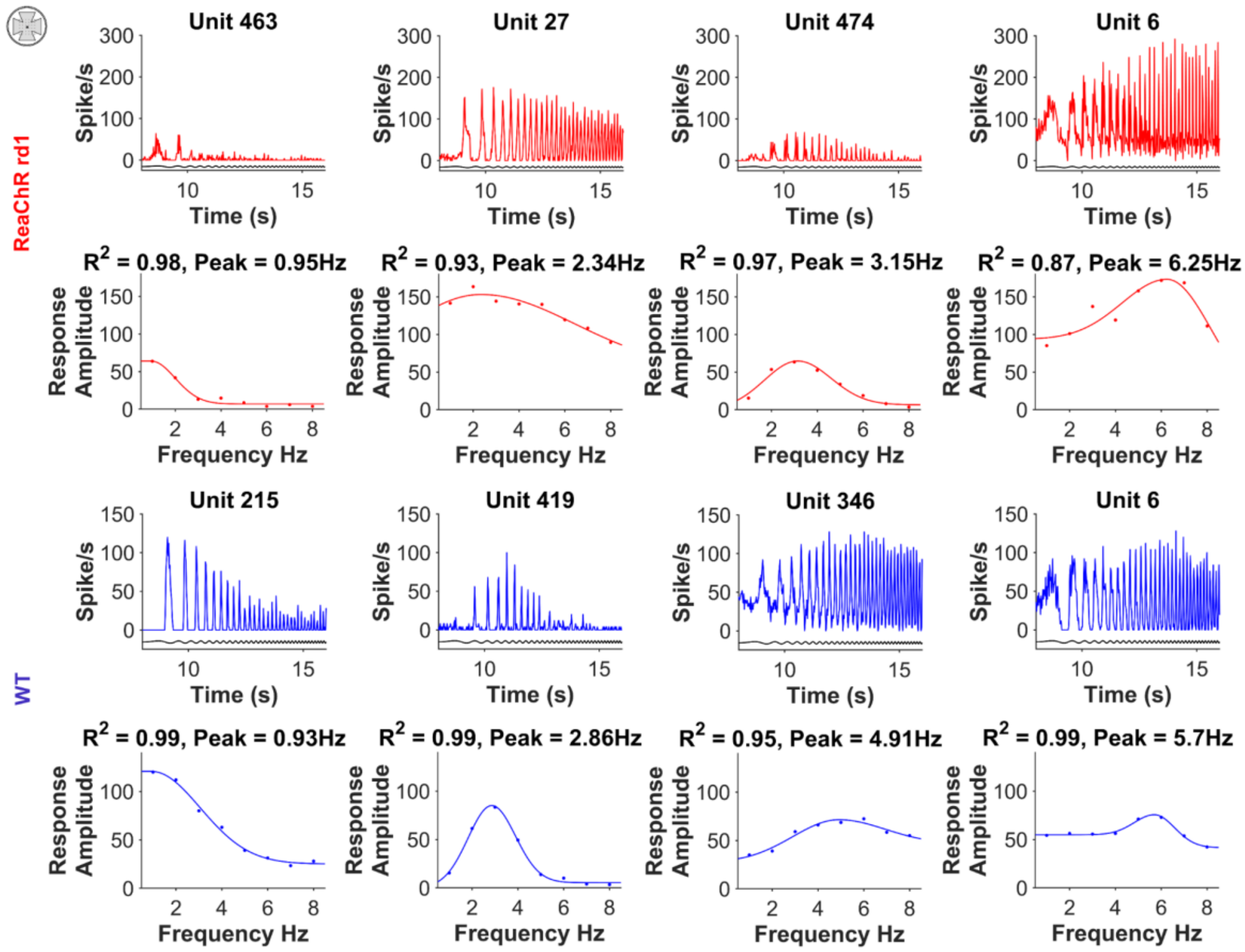

Figure S3. Diverse temporal frequency tuning in *ReaChR rd1* and WT retina
Related to Figure 4

Time course (top) and temporal frequency tuning function (bottom row) for 4 example units from a) *ReaChR rd1* (red) and b) WT (blue) retinas, showing diversity in peak frequency and bandwidth of tuning curves within each genotype.

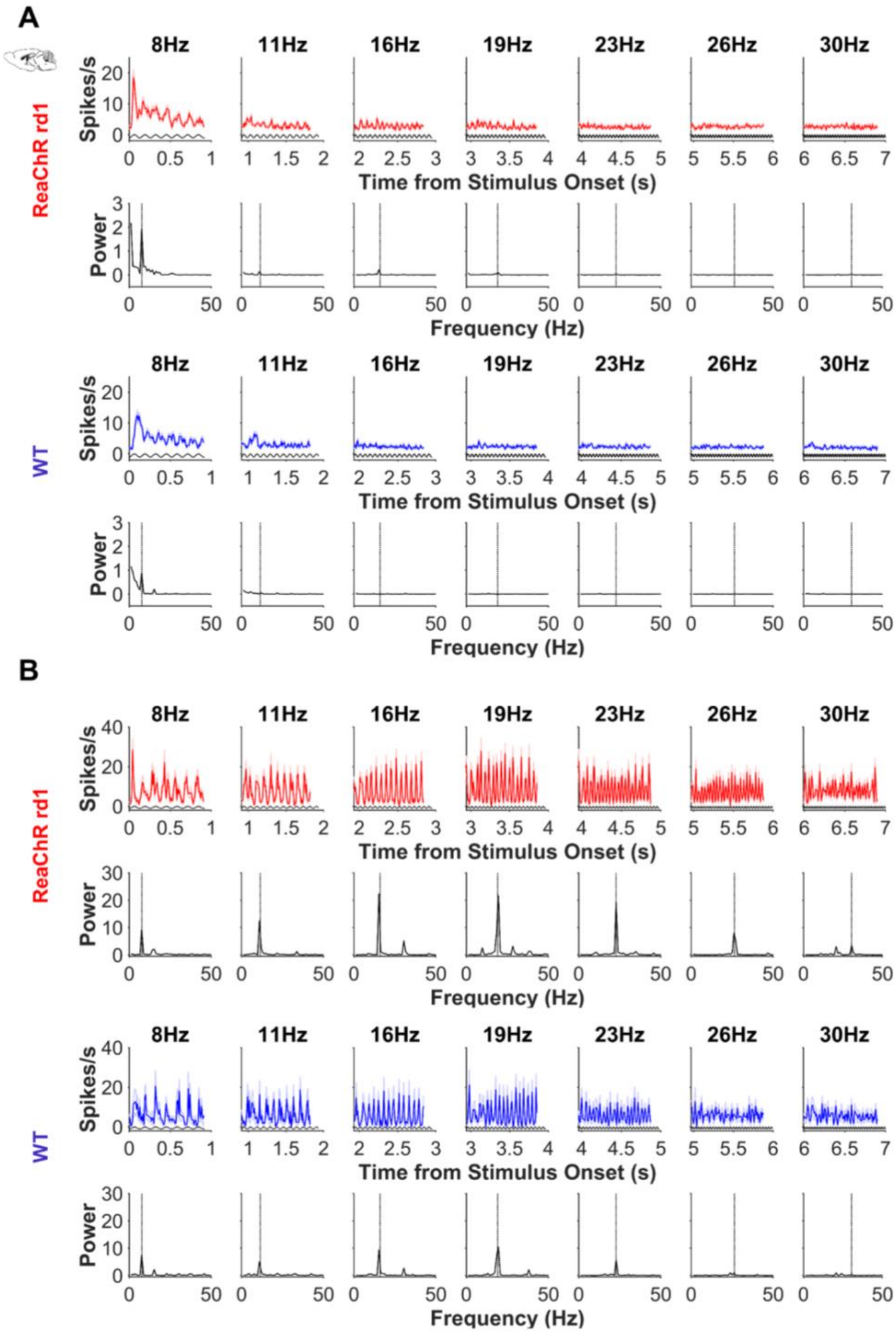

Figure S4. Temporal frequency tuning in the *ReaChR rd1* and WT dLGN
Related to Figure 4

Mean PSTH (top, 10ms bins) and periodogram (bottom) at each stimulus frequency (labelled above PSTH) for all dLGN units with peak power at either

a) 8Hz (N = 199 for *ReaChR rd1* in red, N = 232 for WT in blue) or

b) 19Hz (N = 43 for *ReaChR rd1* in red, N = 19 for WT in blue). Stimulus timing shown in black on PSTH. Dotted vertical line on periodogram shows stimulus frequency.

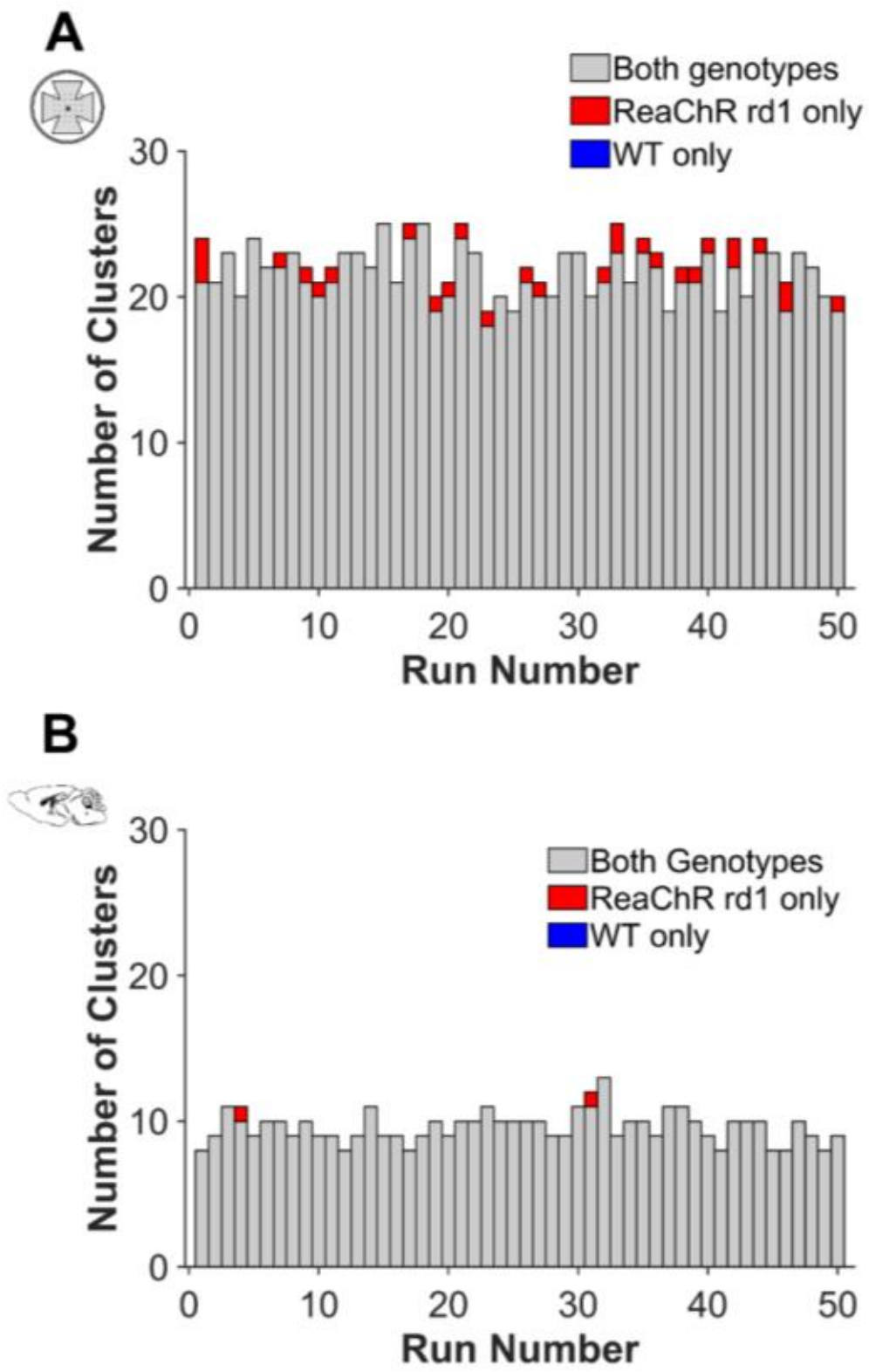

Figure S5. Summary of 50 runs of probabilistic clustering
Related to Figure 6

Summary of 50 runs of clustering based on Gaussian Mixture Models (Baden et al., 2016). Bar heights shows the total number of clusters generated for each repeat of clustering algorithm, with different colours indicating the relative number of clusters that contain units from both genotypes (grey), *ReaChR rd1* units only (red) or WT units only (blue). Shown for a) retina and b) dLGN data.

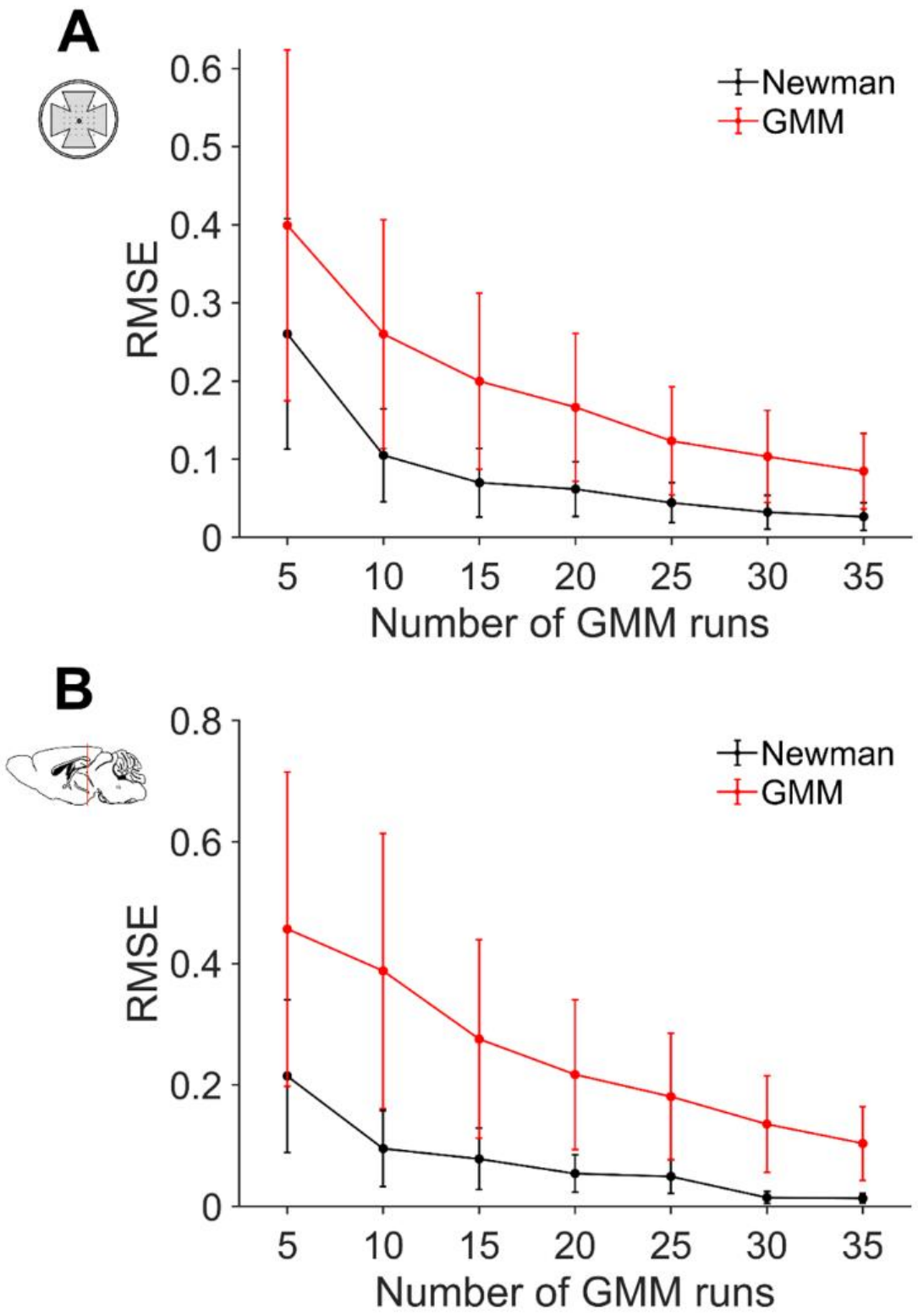

Figure S6. Community detection increases the reliability of probabilistic clustering Related to Figure 6

a-b) Root mean square error (RMSE, Mean±Standard Deviation) across similarity matrices obtained by sampling an increasing number of repeats of the Gaussian Mixed Models (GMM) clustering algorithm (red lines). RMSE decreases as function of the number of GMM repeats sampled, reported on the x-axis. At each repeat of the clustering algorithm, the associated similarity matrix was also employed to detect communities by using Newman community detection[S2, S3], and a new similarity matrix was generated based on such communities. RMSE (Mean±Standard Deviation) was then recalculated based on the Newman-derived similarity matrices (black lines). Note that the application of community detection (black lines) substantially reduces the RMSE. Shown for a) retina and b) dLGN data.

Supplemental References

S1. Euler, T., Haverkamp, S., Schubert, T., and Baden, T. (2014). Retinal bipolar cells: elementary building blocks of vision. Nat. Rev. Neurosci. *15*, 507–519. 10.1038/nrn3783.

S2. Newman, M.E.J. (2006). Modularity and community structure in networks. Proc. Natl. Acad. Sci. U. S. A. *103*, 8577–8582. 10.1073/pnas.0601602103.

S3. Rubinov, M., and Sporns, O. (2010). Complex network measures of brain connectivity: uses and interpretations. Neuroimage *52*, 1059–1069. 10.1016/j.neuroimage.2009.10.003.
